# Metformin enhances methylene blue-mediated photodynamic therapy in oral squamous cell carcinoma

**DOI:** 10.1186/s43046-026-00359-6

**Published:** 2026-06-22

**Authors:** Mohamed A. Osman, Marwa Sharaky, Hesham Abdel-Fattah, Ali M. Safaan

**Affiliations:** 1 Dental Unit, Surgical Oncology Department, National Cancer Institute, Cairo University, Cairo, Egypt; 2 Cancer Biology Department, Pharmacology Unit, National Cancer Institute, Cairo University, Cairo, Egypt; 3 Department of Medical Application of Laser, National Institute of Laser Enhanced Sciences, Cairo University, Cairo, Egypt

**Keywords:** Metformin, Methylene Blue-mediated PDT, Tongue Squamous Cell Carcinoma, AMPK, mTOR

## Abstract

**Background:**

Head and neck squamous cell carcinoma (HNSCC) is the most common malignancy of the head and neck region with relatively low survival rate and response and high resistance to treatment. Tongue and floor of the mouth are the most commonly involved sites. New therapies for oral squamous cell carcinoma (OSCC) treatment are still required. An attractive option for the treatment of OSCC is the use of photodynamic therapy (PDT). Considering metformin’s potential to reduce cancer risk and inhibit proliferation, therefore the present study aims to evaluate its ability to potentiate the methylene blue-mediated photodynamic effect against the growth of tongue cancer cells in vitro and the possible molecular mechanisms of action involved.

**Methods:**

Cytotoxic activity of metformin (MF) and/or Methylene blue (MB)+laser light (L) against the growth of HNO97 tongue cells was determined using the sulforhodamine (SRB) method. Several protein expressions were determined using ELISA kits include mechanistic target of rapamycin (mTOR), AMP-activated protein kinase (AMPK), B-cell lymphoma 2 (Bcl-2), Bcl-2-associated X protein (Bax), and caspase 8.

**Results:**

Laser light at different energy per surface area induced inhibition in the surviving fraction of HNO97 cells, while in presence of MB (10 µg/ml for 4 h.), the surviving fractions showed more inhibition. Addition of MF (1000 µg/ml, for 24 h.) to MB + L induced significant 51 and 89% inhibition of cell survival at energy level of 45 and 337.5 J/cm^2^, respectively. Addition of Metformin to MB-photodynamic therapy also increased the expression AMPK and decrease the expression of mTOR at the protein level. Moreover there were increase in Bax expression and decrease in Bcl2 expression with significant increase in caspase 8 expression at the protein level.

**Conclusion:**

Such combined treatment showed promising synergistic interaction via several molecular pathways, which is well tolerated and readily applicable strategy to improve the therapeutic outcome of PDT in cancer cell treatment.

## Introduction

Oral squamous cell carcinoma (OSCC) is among the most prevalent head and neck malignancies, characterized by aggressive local invasion, late diagnosis, and limited therapeutic success despite advances in surgery, radiotherapy, and chemotherapy [1]. Conventional treatments—including surgery, radiotherapy, and chemotherapy are often associated with significant morbidity, impaired quality of life, and limited effectiveness in advanced disease stages [2]. Methylene blue–mediated photodynamic therapy (MB-PDT) has demonstrated significant cytotoxicity against OSCC cells, yet its therapeutic potential may be restricted by tumor cell redox capacity, mitochondrial function, and metabolic adaptability [3].Although direct studies investigating the combination of PDT and metformin (antidiabetic biguanide) in oral squamous cell carcinoma are limited, substantial evidence from OSCC and related squamous cell carcinoma support our approach. Metformin (antidiabetic drug),, has gained wide attention as a repurposed anticancer agent due to its ability to modulate cellular metabolism, inhibit mitochondrial complex I, reduce ATP production, attenuate oxidative stress, and trigger AMPK activation [4–6]. Importantly, metformin has been shown to enhance tumor sensitivity to other reactive oxygen species ROS-inducing therapies, including ionizing radiation and chemotherapeutics, by impairing DNA repair, exacerbating metabolic stress, and promoting apoptosis [7, 8]. Through these metabolic effects, metformin increases cellular susceptibility to oxidative stress while lowering ATP levels and impairing survival pathways that support tumor resistance. Combining metabolic modulators with PDT has recently been proposed to enhance phototoxic outcomes by overwhelming cancer cells antioxidant defenses [9]. Therefore, elucidating the molecular mechanism through which metformin potentiates MB-PDT may contribute to developing more effective combination therapies for OSCC. So, The current study hypothesizes that metformin may potentiate the cytotoxic activity of MB-mediated PDT against the growth of oral squamous carcinoma cells (HNO97) via modulation of AMPK/mTOR signaling and apoptosis pathways.

## Materials and methods

### Drugs

Methylene blue (MB) and MF were purchased from Sigma-Aldrich (St. Louis, MO, USA). Both compounds were diluted in PBS and filtered with a 0.22 μm syringe filter. Both compounds were stored in at -20 °C freezer until use.

### Cells and cell culture

Human tongue squamous cell carcinoma cell line (HNO97) obtained from the American Type Culture Collection (ATCC, Manassas, VA, USA). The cells was cultured in DMEM Dulbecco’s modified Eagle’s medium (Gibco, UK) to which penicillin (100 I.U. /ml) and streptomycin (100µg/ml) had been added. The medium was supplemented with 10% fetal bovine serum (FBS; Gibco, UK) and the cells were maintained in a humidified incubator at 37 °C and 5% CO2.

### Laser light

Diode laser (Woodpecker LX 16 plus, China)) at wave length 650 nm, 120 mW power and different energy density ranging from 45 to 337.5 J/cm^2^ have been used in this study. Each well was irradiated with the sweeping motion of the laser’s hand piece and tip (DT30-Tip) [with tip physical size approximately 68.7 × 32 mm] for 2 to20 minutes in a continuous-wave mode and the distance between the laser hand piece and the well was 1 mm.


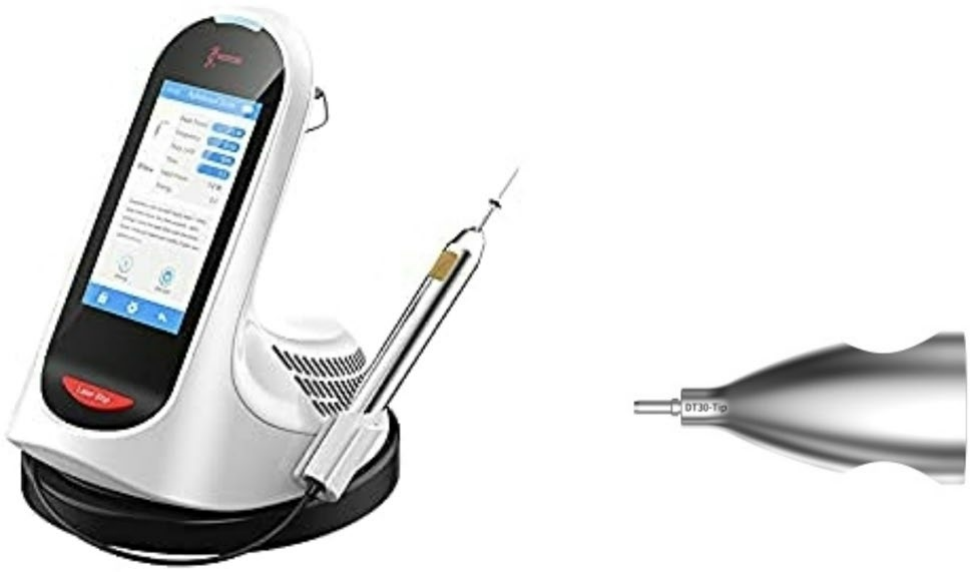


### Assessment of inhibitory effect of MB-mediated PDT in presence of MF against the survival of HNO97 cells

Cytotoxic activity of metformin and/or Methylene blue+laser light was determined using the sulforhodamine (SRB) method as previously prescribed by Skehan et al. [10]. In brief, HNO97 cells were plated in 96 well micro plates at a concentration of 5 × 10^3^ cells/well in DMEM Dulbecco’s modified Eagle’s medium. After 24 h. Incubation period, cells were treated with MF (1000µg/ml) for 20 h. and then add MB (10 µg/ml ,4 h.) alone in separate wells or in combination with metformin and continue incubation till 24 h. Cells were exposed to laser light at different energy ranging from 45 to 337.5 J/cm^2^ and re-incubated for another 24 h. Then, cells were fixed by adding 50 µl of cold 50% TCA for 1 h.at 4 °C, supernatant was discarded and the wells were washed with distilled water, air dried, stained for 30 min at room temperature with 0.4% SRB dissolved in 1% acetic acid and then washed with 1% acetic acid. The plates were then allowed to air dried, and the dye was dissolved in 100 µl/well of 10 mM Tris base (pH 10.5) for 10 min. Each well’s optical density (OD) was measured spectrophotometrically at 490–530 nm using an ELISA microplate reader (TECAN Sunrise TM, Germany), with automated shaking taking place for 30 s before reading.

The percent inhibition was calculated using sigmoidal dose response curve-fitting models (Graph Pad, Prizm8.02 software incorporated).

### Determination of mTOR and AMPK concentrations in HNO97 cell lysate

mTOR and AMPK concentrations were measured spectrophotometrically at 450 nm in treated and control cell lysate using ELISA Assay Kits (Elabscience^®^ Human ELISA Kit, Houston, Texas, 77079, USA, Catalog No.: EH1686, and EH2622 ,respectively following the manufacturer’s instructions.

### Determination of Bcl-2 and Bax concentrations in HNO 97 cell lysate

BCL2 and BAX concentrations were measured spectrophotometrically at 450 nm in treated and control cell lysate using ELISA Assay Kits (Elabscience^®^ Human ELISA Kit, Houston, Texas, 77079, USA, Catalog No. EH0658, and EH0669), respectively following the manufacturer’s instructions.

### Assay of caspase 8

The apoptotic activity was determined by measuring the protein concentrations of caspase 8 spectrophotometrically at 450 nm in cell lysate using ELISA Assay Kits (Elabscience^®^ Human ELISA Kit, Houston, Texas, 77079, USA, Catalog No. Catalogue No.: EH0682) following the manufacturer’s instructions.

### Statistical analysis

All data were expressed as the mean **±** standard deviation of the mean (S.D.M.). The statistical analysis was performed using a parametric repeated measure one-way analysis of variance (ANOVA) followed by Tukey honest significant difference (HSD) test using the statistical package for the social sciences (SPSS) software (version 18.0, Chicago, IL, USA). Statistical significance.

was set at a level of *p* < 0.05.

### Ethics statement

All the in vitro study protocols and studies were approved by the Institutional review board of National Cancer Institute, Cairo University, number SO2506-110-2005 and National Institute of Laser Enhanced Science, Cairo university Number 25-6-17 (in).

## Results

### Inhibitory effect of methylene blue -mediated photodynamic therapy on survival of Tongue HNO97 cells in presence of metformin

**Fig. 1:**
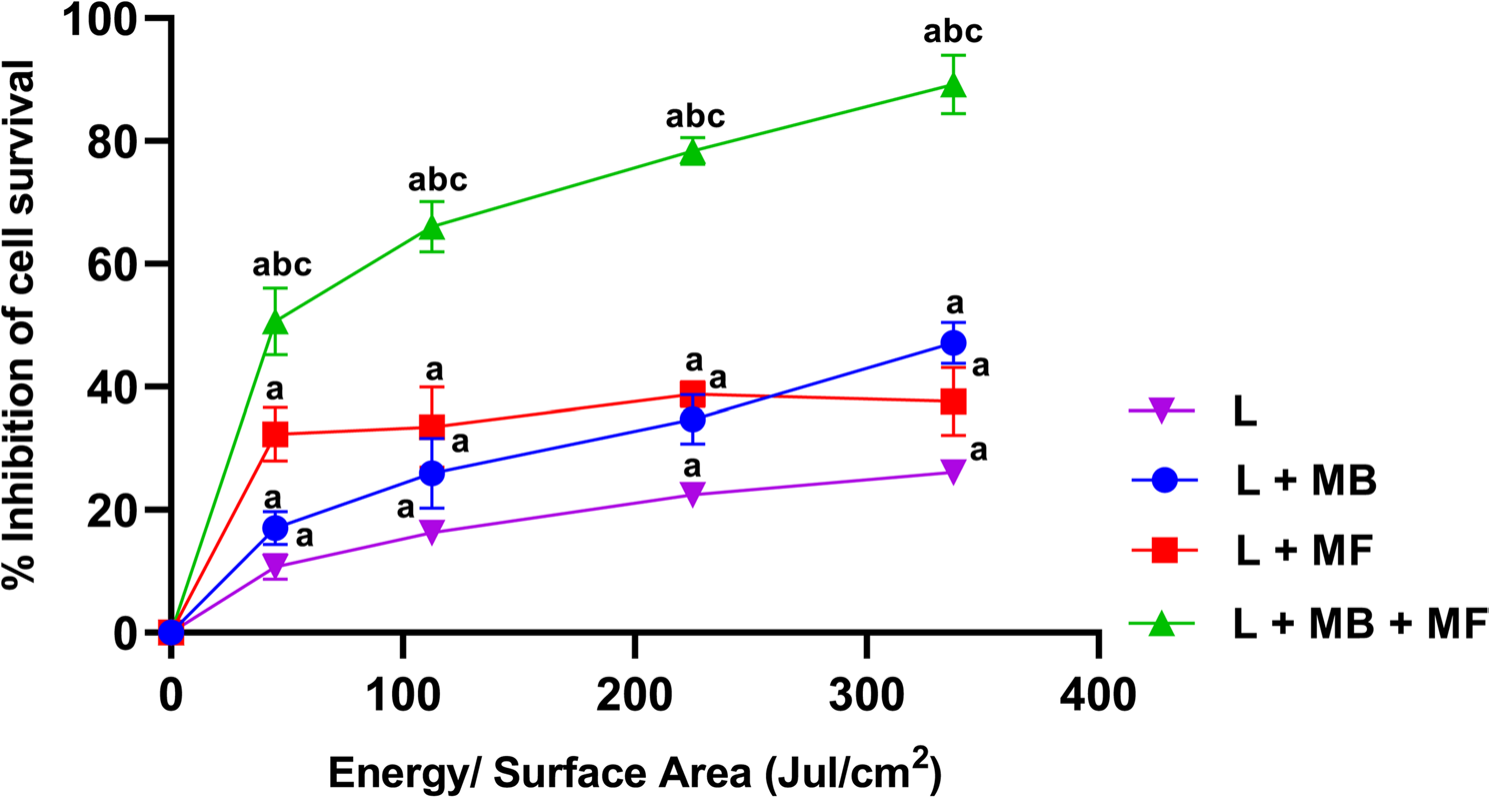
Inhibitory effect of MB-mediated PDT on survival of HNO97 cells in presence of MF. HNO97 cells were plated in 96 well micro plates at a concentration of 5×10^3^ cells/well in DMEM Dulbecco’s modified Eagle’s medium. After 24 hrs. incubation period ,cells were treated with MF (1000 µg/ml) for 20 hrs. and then add MB (10 µg/ml ,4 hrs.) alone in separate wells or in combination with metformin and continue incubation till 24 hrs. Cells were exposed to laser light at different energy ranging from 45-337.5 J/cm^2^ and re-incubated for another 24 hrs. and continue as mentioned in the materials and methods. All data were expressed as the mean ± standard deviation of the mean (S.D.M.) for 2 independent experiment 3 -4wells each ^a^ significant from laser light at *p*<0.01, ^b^ significant from MB+L at *p*<0.01, ^c^ significant from MF+L at *p*<0.01

**Fig. 2:**
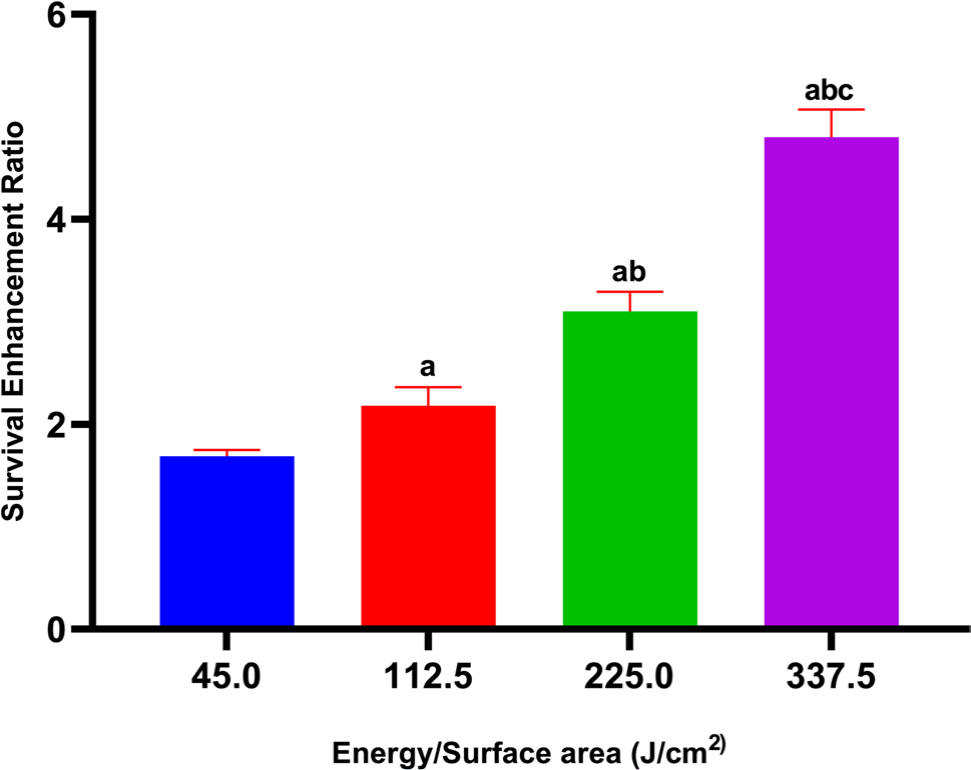
Survival enhancement ratio of methylene blue (MB, 10 µg/ml,4 hrs.) mediated- photo dynamic therapy in presence of metformin (MF,1000 µg/ml, 24 hrs.). Legend as in Fig. 1. Energy/surface area = 0.12W X time of exposure per second÷0.32. Survival Enhancement Ratio = Surviving fraction of (MB+L) ÷ surviving fraction of (L+MB+MF). All data were expressed as the mean ± standard deviation of the mean (S.D.M.) for 2 experiments 4 wells each. If ˃ 1 = increase photodynamic therapy, if 1 = No effect, if ˂ 1 = protective effect. Each point is the mean ± S.D.M. of two experiments, each one in quadruplicates, ^a^ significant from first dose at *p*<0.05.^b^ significant from first and second dose at *p*<0.05. ^c^ significant from all doses at *p*<0.01

Figure 1 showed that laser light at different energy per surface areas induced decrease in the surviving fraction of HNO97 cells showed about 10 to 26% inhibition of cell survival at energy level of 45 and 337.5 J/cm^2^, respectively. While in presence of MB (10 µg/ml for 4 h.), the surviving fractions have been decreased by 17 and 47%. Addition of MF (1000 µg/ml, for 24 h.) to MB + L induced significant 51 and 89% inhibition of cell survival, respectively. Figure 2 shows that MF induced sensitization of tongue cells to the action of MB-mediated photodynamic treatment ranging from 1.69 to 4.8 at energy levels of 45 to 33.7.5 J/cm^2^, respectively. Where more than 1 means synergistic increase in photodynamic therapy.

### Effects of metformin and/or methylene blue-mediated photodynamic therapy on AMPK and mTOR expression at the protein level of tongue cancer cells HNO97

**Fig. 3:**
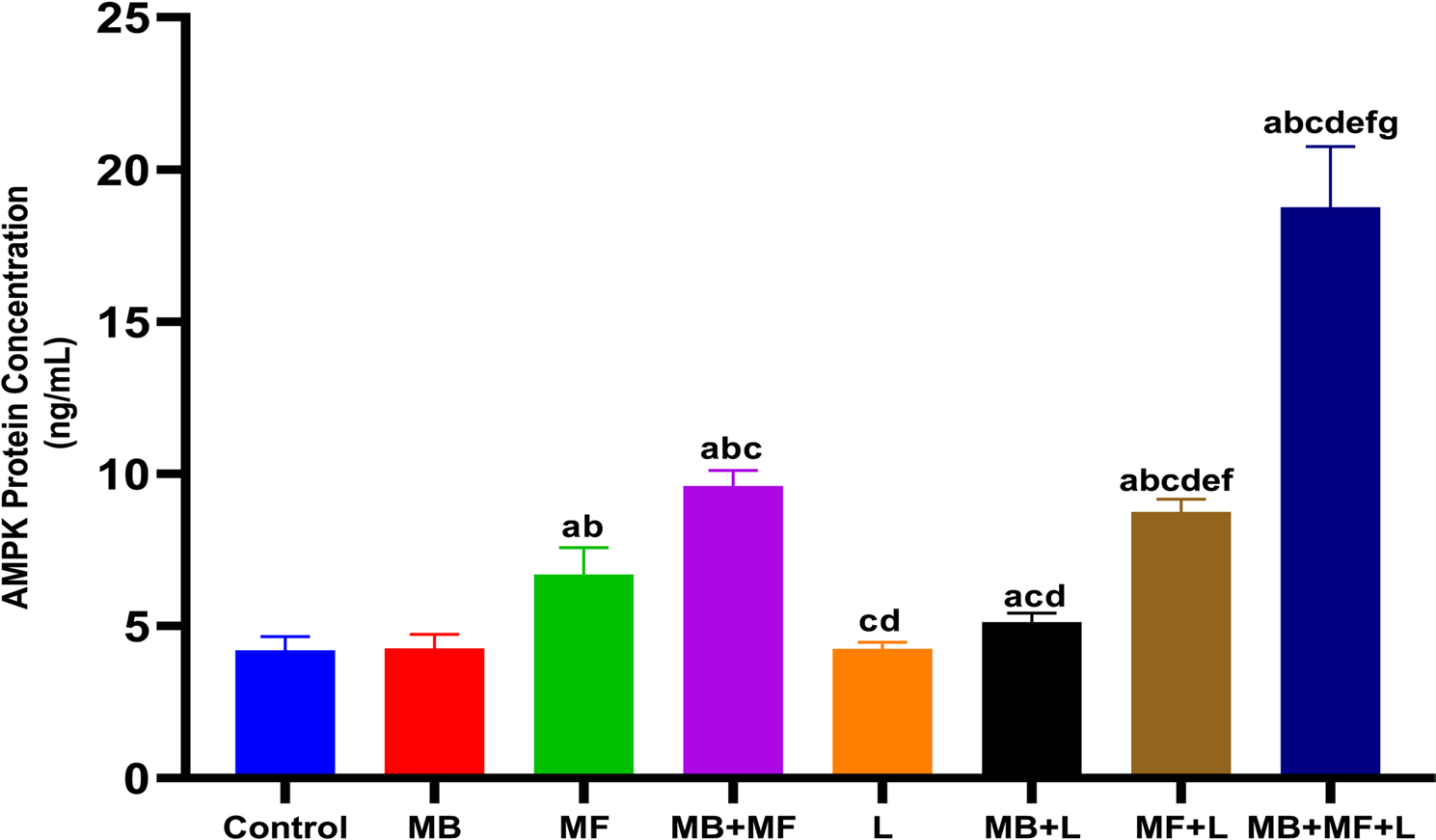
Effects of metformin and/or methylene blue-mediated photodynamic therapy on AMPK expression of tongue cancer cell line HNO97. All data were expressed as the mean ± standard deviation of the mean (S.D.M.) for 2 experiments 3 wells each. ^a^ significant from control at *p*˂ 0.05, ^b^ significant from MB at *p*˂ 0.01, ^c^ significant from MF at *p*˂ 0.01, ^d^ significant from MB+MF at *p*˂ 0.01, ^e^ significant from L at *p*˂0.01, ^f^ significant from MB+L at *p*˂ 0.01, ^g^ significant from MF+L at *p*˂0.01

**Fig. 4:**
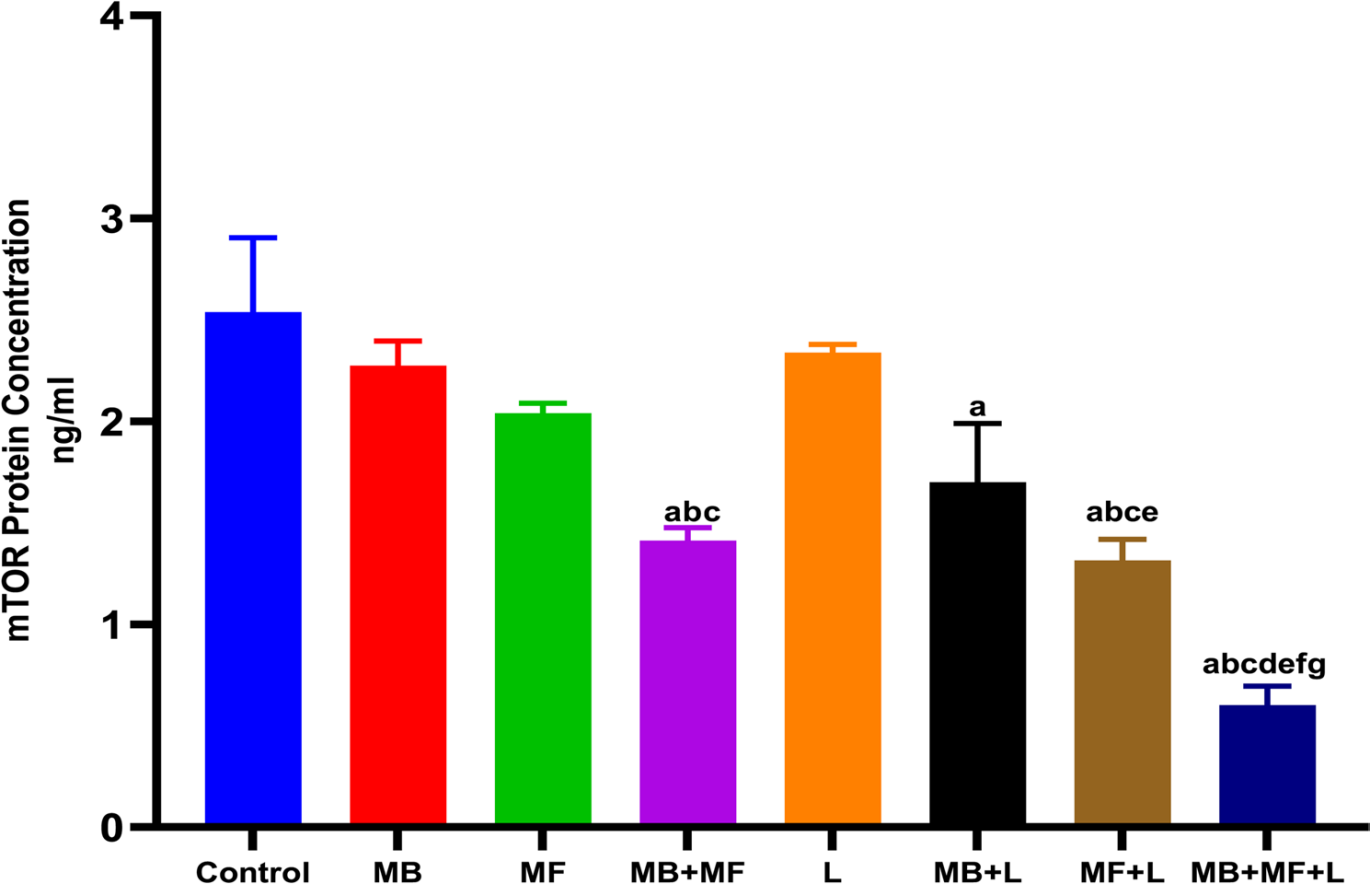
Effects of metformin and/or methylene blue-mediated photodynamic therapy on mTOR expression of tongue cancer cell line . All data were expressed as the mean ± standard deviation of the mean (S.D.M.) for 2 experiments 3 wells each ^a^ significant from control at *p*˂ 0.05, ^b^ significant from MB at *p*˂ 0.01, ^c^ significant from MF at *p*˂ 0.01, ^d^ significant from MB+MF *p*˂0.05, ^e^ significant from L at p< 0.01, ^f^ significant from MB+L at *p*˂ 0.01, ^g^ significant from MF+L at *p*˂0.01HNO97

**Fig. 5:**
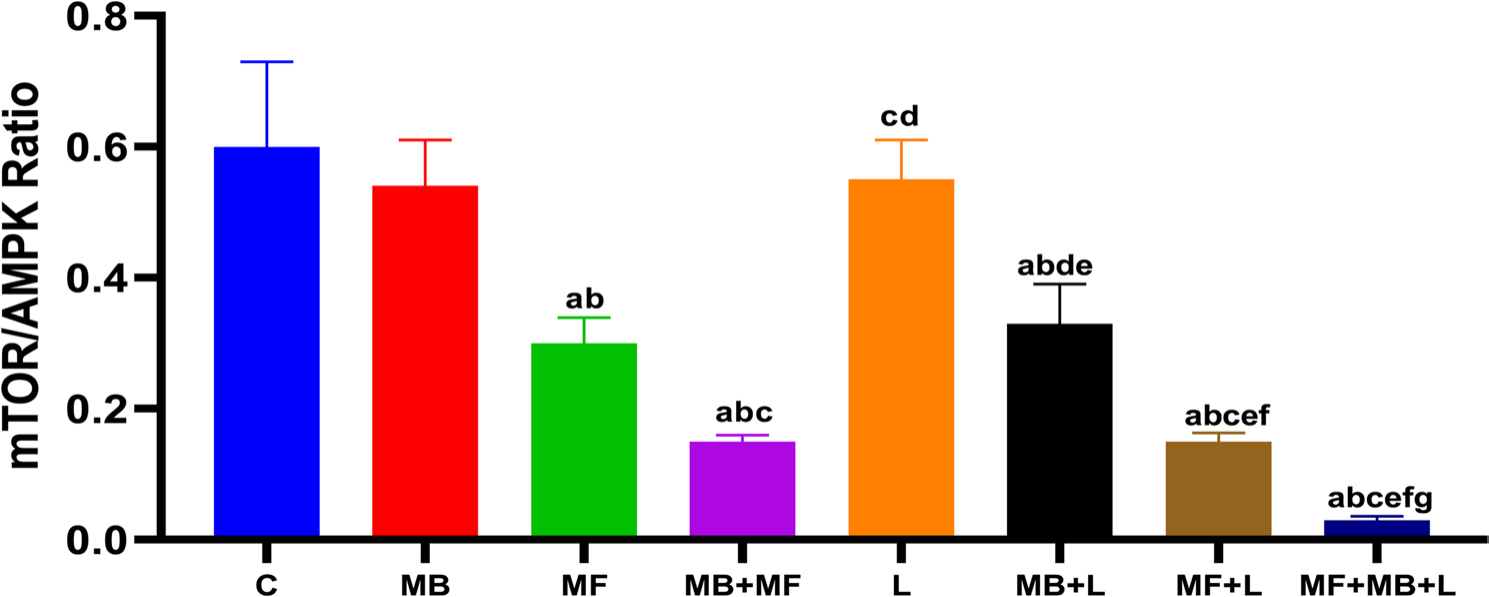
Effects of metformin and/or methylene blue-mediated photodynamic therapy on AMPK/mTOR ratio in tongue cancer cell line HNO97 . All data were expressed as the mean ± standard deviation of the mean (S.D.M.)for 2 experiments 3 wells each. ^a^ significant from control at *p*˂ 0.05, ^b^ significant from MB at *p*˂ 0.01, ^c^ significant from MF at *p*˂ 0.01, ^d^ significant from MB+MF, ^e^ significant from L at *p*˂0.01, ^f^ significant from MB+L at *p*˂ 0.01, ^g^ significant from MF+L at *p*˂0.01

Figures 3, 4 and 5 show the effect of MB mediated –photodynamic therapy on expression of AMPK and mTOR at the protein level in HNO97 tongue cells in presence and absence of Metformin .Laser beam did not show changes in AMPK expression compared to control, but in presence of MB and MF there were high expression [about 1.22 and 11.8 fold, respectively).Addition of MF to MB- mediated photodynamic therapy induced high significant increase in AMPK expression compared to Control, L, MB + L, MF + L [4.48, 4.42, 3.65 and 2.16 fold, respectively). On the other hand, MB-mediated photodynamic therapy (MB + L) showed decrease in expression of mTOP by about 33% compared to control but MF + L showed more decrease in the expression (48%) compared to control. Addition of MF to MB-mediated photodynamic therapy (MB + L) induced high significant decrease compared to control, MB + L, MF + L (33, 65, 54%, respectively).

Ratio of mTOR/AMPK which reflects the balance between growth suppression and stimulation appeared in Fig. 5 showed that addition of MF to MB-mediate photodynamic therapy (MB + L) induced significant low value ( about 0.03) compared to control, L, MB + L, MF + L (0.60, 0.55, 0.33, 0.15,respectively).which represented a high significant decrease in cell growth.

### Effects of metformin and/or methylene blue-mediated photodynamic therapy on Bcl2 and Bax expression at the protein level of tongue cancer cells HNO97

**Fig. 6:**
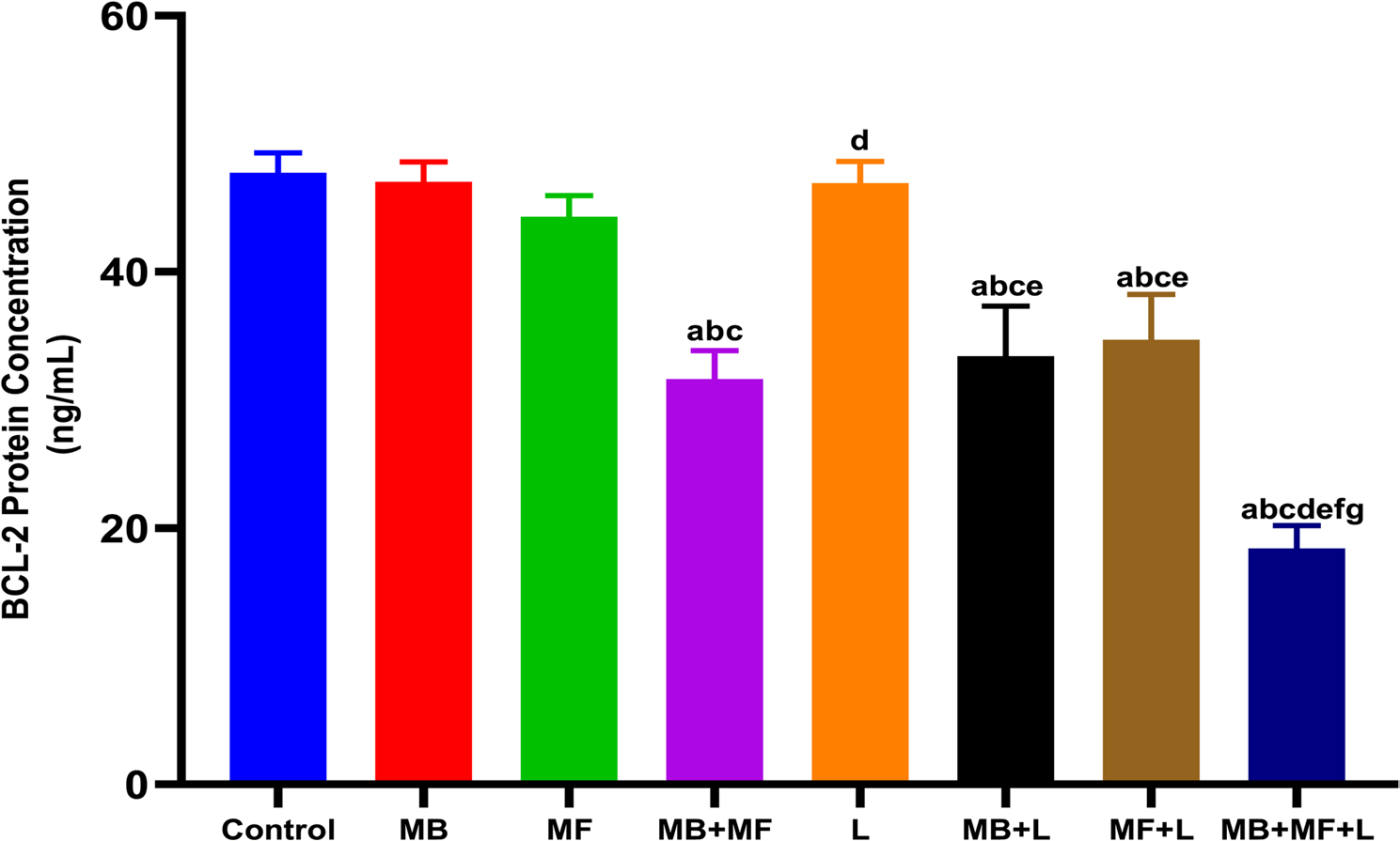
Effects of metformin and/or methylene blue-mediated photodynamic therapy on Bcl2 expression of tongue cancer cell line HNO97. All data were expressed as the mean ± standard deviation of the mean (S.D.M.)for 2 experiments 3 wells each. ^a^ significant from control at *p*˂ 0.05, ^b^ significant from MB at *p*˂ 0.01, ^c^ significant from MF at *p*˂ 0.01, ^d^ significant from MB+MF at *p*˂0.05, ^e^ significant from L at *p*˂ 0.01, ^f^ significant from MB+L at *p*˂ 0.01, ^g^ significant from MF+L at *p*˂0.01

**Fig. 7:**
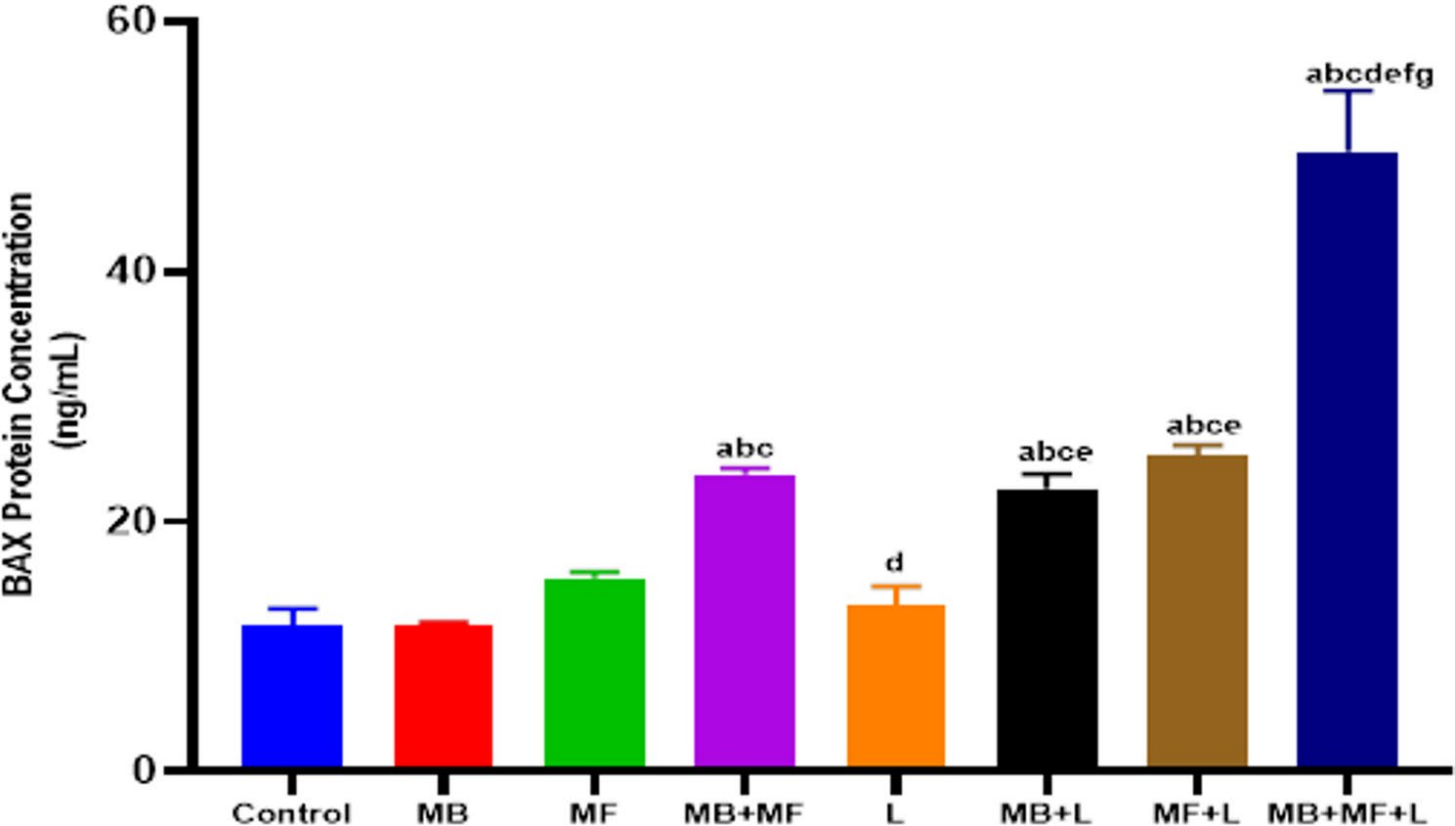
Effects of metformin and/or methylene blue-mediated photodynamic therapy on Bax expression of tongue cancer cell line HNO97. All data were expressed as the mean ± standard deviation of the mean (S.D.M.)for 2 experiments 3 wells each. ^a^ significant from control at *p*˂ 0.05, ^b^ significant from MB at *p*˂ 0.01, ^c^ significant from MF at *p*˂ 0.01, ^d^ significant from MB+MF at *p*˂0.01, ^e^ significant from L at *p*<0.01,^f^ significant from MB+L at *p*˂ 0.01, ^g^ significant from MF+L at *p*˂0.01

**Fig. 8:**
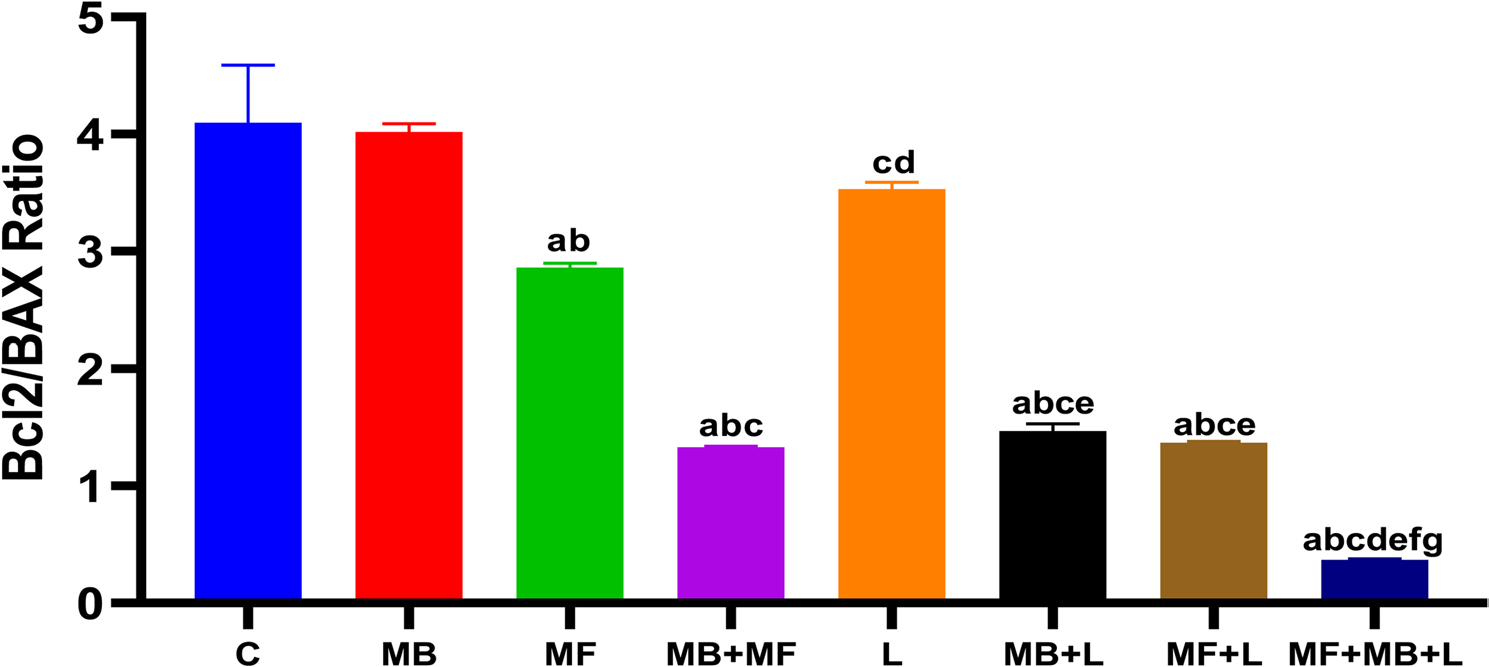
Effect of laser light (Energy / surface area) on Bcl2/Bax ratio in HNO97 cells in presence and absence of Methylene blue (10 µg/ml, 4 hr), and Metformin (1000 µg/ml, 24 hr). All data were expressed as the mean ± standard deviation of the mean (S.D.M.)for 2 experiments 3 wells each. ^a^ significant from control at *p*˂ 0.05, ^b^ significant from MB at *p*˂ 0.01, ^c^ significant from MF at *p*˂ 0.01, ^d^ significant from MB+MF at *p*˂0.05, ^e^ significant from L at *p*˂0.01, ^f^ significant from MB+L at* p*˂ 0.01,^g^ significant from MF+L at *p*˂0.01

**Fig. 9:**
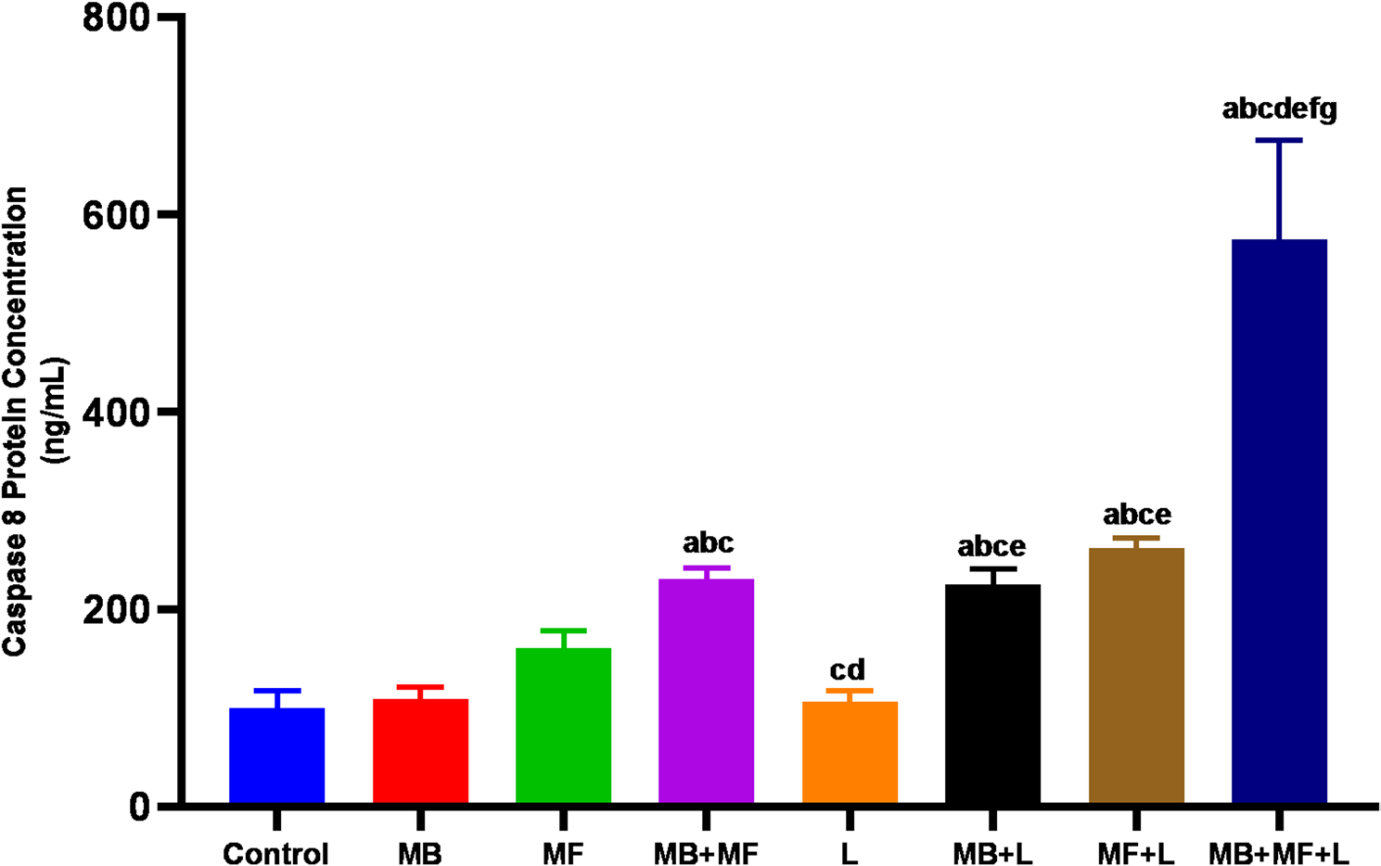
Effects of metformin and/or methylene blue-mediated photodynamic therapy Caspase 8 expression in tongue cancer cell line HNO97 All data were expressed as the mean ± standard deviation of the mean (S.D.M.)for 2 experiments 3 wells each. ^a^ significant from control at *p*˂ 0.05, ^b^ significant from MB at *p*˂ 0.01, ^c^ significant from MF at *p*˂ 0.01, ^d^ significant from MB+MF at *p*˂0.01, ^e^ significant from L at *p*˂0.01, ^f^ significant from MB+L at *p*˂ 0.01, ^g^ significant from MF+L at *p*˂0.01

Figures 6, 7 and 8 show the effect of MB-mediated PDT on expression of Bcl2 and Bax at the protein level in tongue HNO97 cells in presence of MF. Laser beam alone did not show significant changes in Bcl2 expression but in presence of MB there was 30% decrease in Bcl2 and 95% increase in Bax expression. Addition of MF to MB + L showed significant decrease in Bcl2 expression at the protein level compared to Control, L, MB + L, MF + L ( 61, 61, 45, 47%, respectively)) .while there was a significant increase in Bax expression by 4.25,3.73, 2.19 and 0.96 fold compared to control, L ,MB + L, MF + L, respectively). Ratio of Bcl2/Bax in figure [9] represents the relation between cell survival and cell death. Laser beam alone did not induce significant changes from control, but addition of MB or MF showed 58 and 61% decrease, respectively. Addition of MF to MB + L showed highly significant decrease in the ratio compare to laser alone ( about 91%)and to MB + L (about 75%).

### Effects of metformin and/or methylene blue-mediated photodynamic therapy on caspase 8 expression at the protein level of tongue cancer cells HNO97

Figure 9 showed that laser alone did not induce significant changes in caspase 8 expression at protein level from control. Addition of MF showed about 117 and 91% increase compared to control and laser alone, respectively. Combination of MF to MB + L induced highly significant increase compared to all groups [about 4.26, 4.23, 3.21,2.09 ,3.73, 2.19 and 0.96 fold, compared to control, MB, MF, MB + MF, L,MB + L, MF + L, respectively].

## Discussion

Oral squamous cell carcinoma is among the most prevalent head and neck malignancies, characterized by aggressive local invasion, late diagnosis and limited therapeutic success despite advances in surgery, radiotherapy and chemotherapy [1].New treatment strategy involve PDT has emerged as minimally invasive modality of selectively inducing cancer cell death through light-activated generation of ROS after photosensitizer excitation [11].In the current study, Addition of the antihyperglycemic metformin increased the lethality of MB-mediated photodynamic therapy (Fig. 1). This has been observed by sensitization of tongue cells to the action of laser + MB by metformin as shown in Fig. 2 where survival enhancement ratio has been increased by increasing the energy in presence of metformin. The Sensitization Enhancement Ratio (SER) is a radiotherapy metric that quantifies the effectiveness of a radiosensitizer by measuring the ratio of radiation doses required to achieve the same cell killing effect with and without the sensitizer agent. An SER value greater than 1.0 indicates that the agent has enhanced the effect of radiation, with higher values representing greater sensitization [12–15]. Although direct studies investigating the combination of PDT and metformin in oral squamous cell carcinoma are limited, substantial evidence from OSCC and related squamous cell carcinoma support our approach. This synergistic effect (Fig. 1) have been confirmed by increase and decrease in the expression of AMKP and mTOR, respectively (Figs. 3 and 4). AMPK and mTOR are important proteins that play complementary roles in metabolic regulation. AMPK is activated during energy-depleted condition to regulate cell growth, proliferation, and metabolism .While mTOR responds to nutritional and hormonal signals to promote protein synthesis [16]. But it is important to note that AMPK activity is primarily regulated by phosphorylation than the total protein expression. So, measurement of total AMPK alone does not fully reflect pathway activation but It has been reported that activation of AMPK activated protein kinase inhibit the mTOR pathways [16] therefore, including mTOR signaling and apoptotic markers may support the involvement of AMPK pathway. In the current study, treatment with Laser beam alone did not induce significant changes in AMPK-activated protein kinase, while addition of MF to the treatment protocol showed 2.1 fold increases in expression of AMPK and 4.25 fold decrease in mTOR expression. This agree with that reported by Goel et al. [17] who showed that MF treatment as antidiabetic drug is presumed to inhibit mTOR expression via stimulation of AMPK. The addition of MF to MB + L induced more decrease in mTOR/AMPK ratio (Fig. 5) indicating suppression of cell growth of HNO97 cells. Moreover, Bcl2 and Bax play a significant role in oral cancer by regulating apoptosis or programmed cell death. It is well known that activation of Bcl2 expression promote s tumor cell survival by inhibiting apoptosis which allows for mutation to accumulate and contributes to resistance to radio or chemotherapy. Conversely, Bax is preapoptotic protein that when decrease or absent, disrupt apoptosis, favoring tumor growth [18] .In the current study, laser alone did not show significant changes from control Bcl2 but addition of MF showed 2.5 fold down regulation in Bcl2 expression and 3.7 fold up regulation in Bax expression with 1.37 ration of Bcl2/Bax compared to 4.1 for control that mean an increase in apoptotic response of MB-mediated PDT in presence of MF [19].This agree with that reported by Raafat et al. [20] where they showed that metformin treatment in nanoparticles induced down regulation of Bcl2 and up regulation of Bax in HEP2 cells which increase the cytotoxic activity of MF against the growth of HEP2 cells. Moreover, Kahllan et al. [21] and Raafat et al. [20] reported down regulation of Bcl2 and up regulation of Bax after PDT that represent a modality for apoptosis. Moreover, addition of MF to MB + L significantly activate caspase-8 expression (Fig. 9) that promotes both extrinsic (via caspase-8) and intrinsic (via BAX-mediated mitochondrial disruption) pathways, resulting in more robust caspase 8 activation and cell death [20].

In conclusion, combing MF with MB-mediated PDT offers a rational strategy to overcome metabolic and signaling-mediated resistance in OSCC.MF imposes energy stress and AMPK-driven mTOR suppression, lowering survival threshold and favoring mitochondrial damage induced by MB-PDT. Also enabling stronger activation of intrinsic (BAX/BCL2 mediated) and potentially extrinsic (caspase-8) apoptotic cascade. This mechanistic rationale supports focused in vitro experiments in OSCC cells to quantify survival, AMPK/mTOR signaling, BCL2/BAX balance and caspase − 8 activation as primary readouts of molecular synergy. Such combined treatment holds promise for well tolerated and readily applicable strategy to improve the therapeutic outcome of PDT in oral cancer treatment. Future study will include multiple tongue derived cell lines, in addition in vivo experiment will be performed to overcome the limitation of just using single tongue cell line and to strengthen and validate the translation relevance of the current study.

## Data Availability

All data generated or analysed during this study are included in this published article (and its supplementary information files).
